# Administrative Dashboard for Monitoring Use of a Web-Based Parent Training Intervention: Usability Study

**DOI:** 10.2196/53439

**Published:** 2024-01-30

**Authors:** Susan M Breitenstein, Julia Berteletti, Shea Smoske, Charles Barger, Kyrie Tipps, Nathan P Helsabeck

**Affiliations:** 1 College of Nursing The Ohio State University Columbus, OH United States; 2 Klein Buendel, Inc Golden, CO United States

**Keywords:** usability, development, dashboard, portal, implementation, design, System Usability Scale, internet-based intervention, parents, parent, child, children, web-based, web-based parent training, PT, descriptive survey, single group, survey, system usability, ezParent, videoconference, information, reviews on usage, improvement, qualitative

## Abstract

**Background:**

Web-based parent training (PT) programs can strengthen parent-child relationships by equipping caregivers with knowledge and evidence-based strategies to manage behavior. Hybrid facilitation of PT includes facilitator interaction paired with self-administered and web-based PT. Web-based administrative dashboards provide users (eg, administrators, facilitators, and researchers) with an integrated platform to monitor parent progress and activities within a PT program or website. Despite the utility and prevalence of administrative dashboards for web-based behavioral interventions, to our knowledge, no research studies have explored the perspectives and insights of dashboard users to enhance user experience and program delivery.

**Objective:**

The purpose of this study is to evaluate the usability of the administrative dashboard (*ez*Dashboard) for the *ez*Parent program, a 6-module web-based PT program for parents of children aged 2-5 years.

**Methods:**

This study used a descriptive, single-group design with administrators who were overseeing the implementation of the *ez*Parent program and trained facilitators for hybrid *ez*Parent delivery. Participants spent at least 30 minutes reviewing and evaluating the *ez*Dashboard and then completed a survey of their experience with the dashboard. The survey included the validated 10-item System Usability Scale and open-ended questions focusing on user performance, navigation ease, and overall usefulness of the *ez*Dashboard.

**Results:**

Participants (N=15) indicated high usability of the *ez*Dashboard with System Usability Scale scoring a total mean score of 83.5 (SD 16.3). Most participants (n=13, 87%) rated the overall user-friendliness of the *ez*Dashboard as *good* (n=3, 20%), *excellent* (n=9, 60%), or *best imaginable* (n=1, 7%). Open-ended questions revealed the *ez*Dashboard is or would be useful to monitor parent progress and trends in engagement (n=8, 53%) and for reviewing topics for discussion and communicating with parents (n=5, 33%). *ez*Parent administrators (n=4) identified that real-time data for *ez*Parent use helps overall management of program uptake. Suggestions for features to add to the *ez*Dashboard included the ability to track partial progress of program modules (4/14, 29%), total time spent per module (2/14, 14%), and exportable reports (4/14, 29%). Other ideas for improvement included direct messaging capabilities, videoconferencing platform integration, and being able to modify participant account and contact information.

**Conclusions:**

Results indicate that the *ez*Dashboard is easy to use and provides functional information to facilitators and administrators in delivering *ez*Parent. Qualitative results indicate that integrating suggested features into the *ez*Dashboard may help provide a smoother experience for facilitators, administrators, and ultimately the parents using the program. Providing resources for facilitators and administrators in real time to monitor intervention participants’ progress in a program can be helpful in tracking progress and providing facilitated support in tailoring program content and program completion.

## Introduction

Parent training (PT) programs—the gold standard for prevention and treatment of child behavior problems [1-3]—aim to strengthen parent-child relationships by providing caregivers with knowledge and evidence-based strategies to effectively strengthen their parenting skills and support their child’s positive behavior. Parents who have participated in PT programs have demonstrated improvements in multiple areas, including improvements in positive parenting skills, self-efficacy, and parent-child interactions; and reductions in negative or harsh parenting and parenting stress [2,4,5]. PT participants’ children exhibit improvements in child behavior, display decreased conduct problems and aggression, improvements in academic performance, enhanced coping skills, and strengthened relationships with both caregivers and peers [2,4]. Traditionally, PT programs have been offered in-person in group or individual settings; however, web-based adaptations have emerged to mitigate the geographic, logistical, and personal barriers of face-to-face delivery [6-8].

In addition to web-based programs, web-based administrative dashboards have emerged as a promising method to improve delivery and management of web-based interventions [9-11]. These dashboards provide an integrated platform to monitor user progress and activities within a program or website. Administrative dashboards are particularly useful tools for administrators, implementers, researchers, and others who require access to detailed information regarding user usage patterns, performance and achievements, and program progress and completion [10,12,13]. For example, in a research and practice context, dashboards can be used to monitor program fidelity and provide clear metrics to understand how the program is being used in real time.

In the context of web-based programs delivering behavioral interventions, administrative dashboards prove particularly valuable. For example, administrative dashboards can include timestamps for login and logout; activity completion; and fill-in responses to in-program prompts, quiz results, page clicks, and diary entries [9,11,14,15]. These program usage metrics enable administrators to make data-driven decisions, identify areas for improvement, and provide critical support to program and research staff when offering personalized guidance to users [10]. Researchers and program facilitators working directly with participants can leverage the dashboard to prepare for discussions by reviewing the participant’s last logins and progress, using fill-in prompt responses to ask tailored questions, and providing troubleshooting recommendations to encourage program engagement [9,11]. Armed with these data points, facilitators can improve their support of participant use of a program, tailor program materials, and ultimately support improvement for child and parent social-behavioral outcomes.

Despite the utility and use of administrative dashboards, to our knowledge, there are no research studies exploring the user experience of an administrative dashboard related to web-based behavioral interventions. Thus, there is an opportunity for further research to optimize existing functionalities and address limitations to better support administrative dashboard users. Usability, which plays a crucial role in the adoption, engagement, and overall effectiveness of web-based administrative dashboards supporting behavioral health interventions, becomes paramount. The usability of a web-based innovation is commonly assessed on several domains including the efficiency, intuitiveness, ease of use, and satisfaction experienced by the user [16]. The purpose of this study was to investigate the usability of the administrative dashboard for the *ez*Parent program, a web-based PT program. Specifically, our goal was to establish a baseline for user performance, ease of navigation, and usefulness.

## Methods

### Study Design

This study was a descriptive, single group survey design with participants who were overseeing the implementation of the *ez*Parent program (administrators) or trained facilitators for hybrid *ez*Parent delivery (facilitators).

### ezParent Program

The *ez*Parent Program is the web-based delivery of the Chicago Parent Program (CPP). The CPP has been shown to be effective in improving positive parenting skills, parenting self-efficacy, and child behavior problems in a population of low-income, urban parents of children aged 2-5 years old [17,18]. The *ez*Parent Program teaches parents and caregivers the evidence-based strategies of CPP using 6 modules that include a video narrator and vignettes of families using the skills, reflection questions, program activities, and in-home practice assignments [19]. In a pilot randomized controlled trial (RCT) of *ez*Parent (n=83), parents completed 82% of the 6 modules, reported high satisfaction with the program, and we found comparable effect sizes in improvements in parenting practices and reductions in parenting stress and child behavior problems to the group-based CPP [20,21]. In an RCT of self-administered *ez*Parent in primary care (n=287), we failed to find significant main effects for parent and child behaviors [22]. Based on our findings and the extant literature suggesting web-based programs are more effective when provided along with human support [22,23] we are currently testing 2 models of hybrid delivery. The first includes 1:1 brief coaching as part of an RCT funded study (see Greene et al [24]) and community-based delivery of *ez*Parent paired with web-based group sessions [25].

### ezParent Dashboard Design and Development

The *ez*Parent program tracks user progress as parents use the program. Custom data tables collect and store user data with timestamps for logins, completed modules, end-of-module surveys, badges, practice assignments, and practice reviews. The administrative dashboard (*ez*Dashboard) was developed to include these data points for hybrid delivery facilitators to monitor parent progress in the program and to inform hybrid sessions to encourage and support parent program uptake.

*ez*Dashboard logins are created internally by programming staff and are provided to administrators and facilitators. Users access a “Parent Lookup” form by entering the unique ID given to parents during *ez*Parent enrollment and the parent’s last name. These form fields are used together to ensure participant data security. The parent is added to the home page or “Parent List” if the user ID and last name match an individual user in the database. The parent list displays the user ID, last name, last login date, and the last viewed module. When a parent has completed their time in the *ez*Parent program, users can easily remove that parent from their home page.

**Figure 1:**
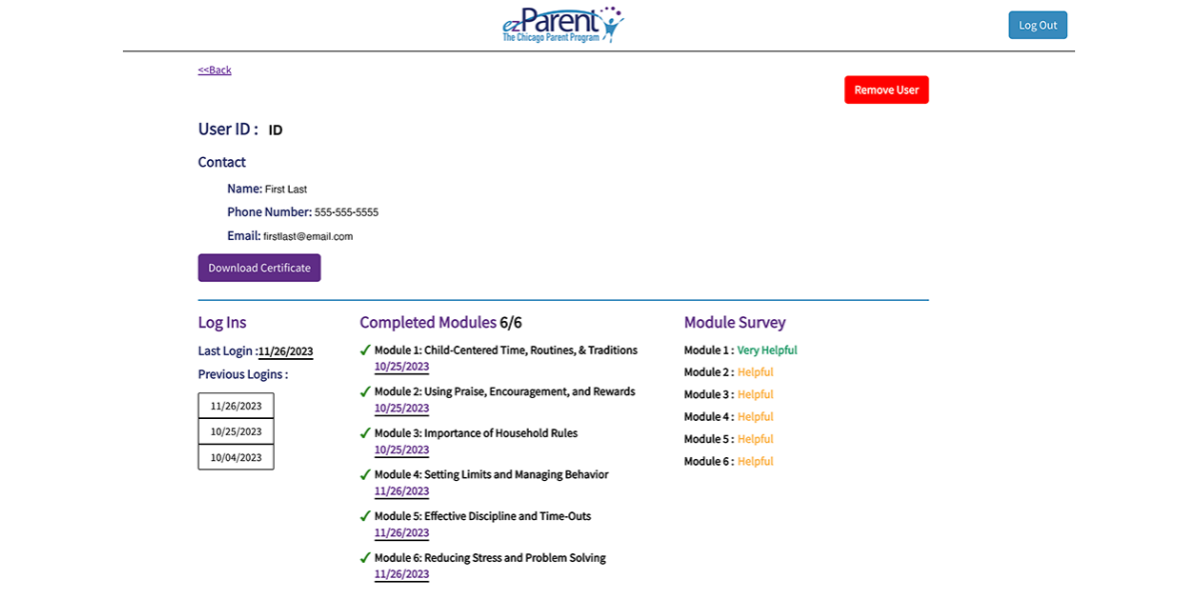
*ez*Dashboard home page: a detailed description of parent use in the program.

**Figure 2:**
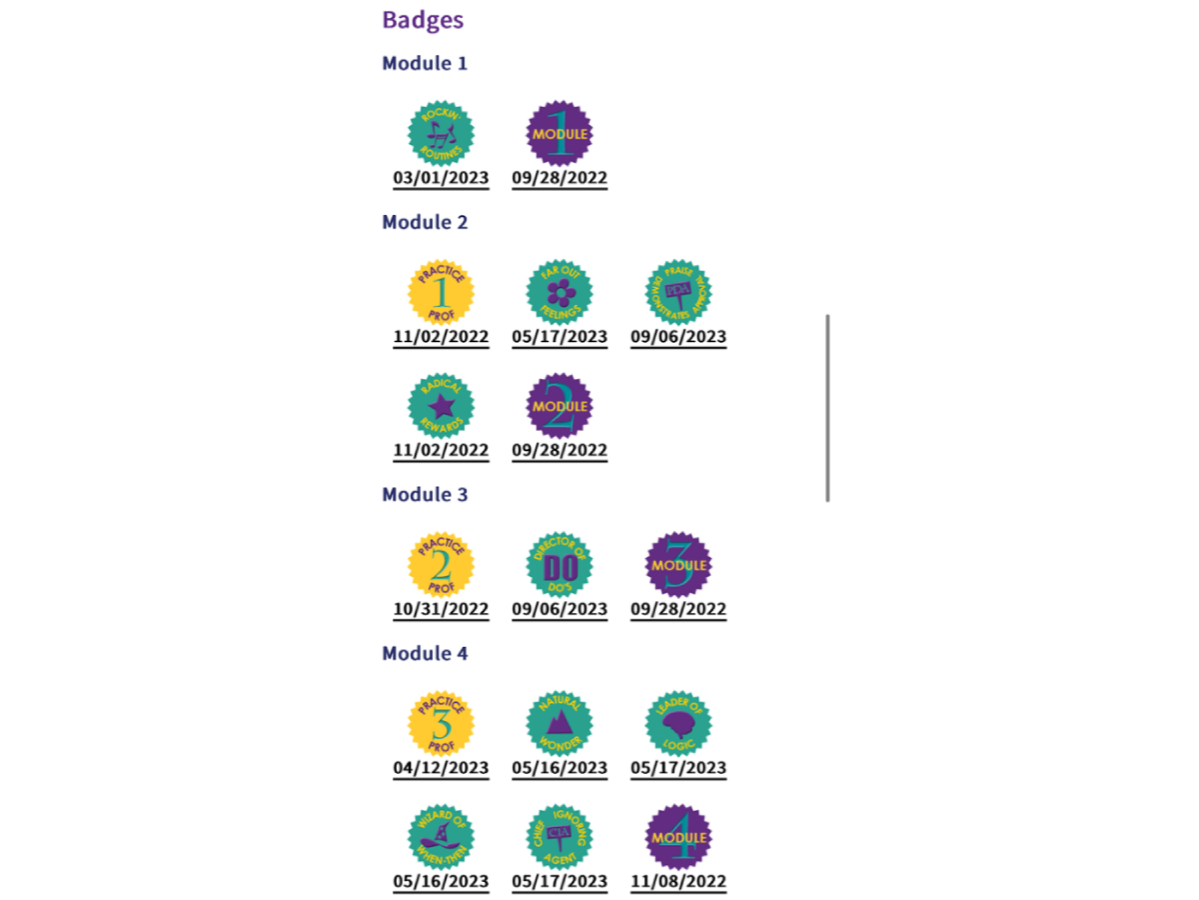
*ez*Dashboard badge completion to mark parent user progress through the program.

From the parent list, *ez*Dashboard users can access more details for an individual parent user by clicking on a “Details” button. The top of the “Details” page displays the user ID, name, phone number, and participant email for easy contact. Contact information is followed by detailed use metrics, including a scrollable list of all login dates, completed modules with a green check mark to indicate completion and the date they were completed, module survey responses, completed badges and dates earned, and responses for practice assignments and practice reviews. Finally, a downloadable, individualized completion certificate is accessible once the last (sixth) module is completed. See Figures 1 and 2 for *ez*Dashboard screenshots.

The *ez*Dashboard’s user interface was made in React (version 17.0.2; MetaOpenSource), using responsive web design principles to allow access across different device sizes. Microsoft SQL Server stores real time user data, which the user interface fetches and displays.

### Ethical Considerations

The research protocol for this study was determined exempt by the institutional review board at The Ohio State University (study number 2023E0289). Participants completed a web-based consent form. All survey data were deidentified and participants were informed that deidentified data may be used or shared without additional informed consent. Participants who consented and completed the survey received a US $25 gift card for participation in this study.

### Participants

We invited *ez*Parent administrators (n=4; individuals overseeing implementation of *ez*Parent) and facilitators (n=19; trained facilitators for hybrid *ez*Parent delivery) to participate in the usability study with a goal for a sample size between 10 and 15. Administrators and facilitators were invited as they may have unique perspectives related to usability depending on their *ez*Parent role. Macefield [26] suggests that a sample size of 3-20 is valid in a usability study to discover usability and potential problems and a sample of 10 will probably reveal a minimum of 82% of the problems and be useful in future design changes. Of the 23 invited to participate in the usability study, 16 consented to participate, and 15 completed the review of the *ez*Dashboard and usability survey.

### Procedures

Potential participants received an email inviting them to participate in the usability study. The email invitation included a description of the project and a link to the consent form in REDCap (Research Electronic Data Capture; Vanderbilt University) [27]. Once consent was obtained, participants were instructed to spend at least 30 minutes reviewing the *ez*Dashboard and evaluating the features. Participants who were not current users of the *ez*Dashboard were provided with login access and instructed to add 2 sample users to their Parent List. Current users of the *ez*Dashboard were instructed to sign into their accounts and review user accounts on their existing Parent List. Both groups were instructed to evaluate their users’ activity on the main page and on the detailed page of all modules in order to evaluate the usefulness of the *ez*Dashboard. These procedures include typical tasks that a facilitator would take in evaluating and monitoring parent use of *ez*Parent. Then, participants were prompted to complete the survey and asked to identify their current role using the ezParent program (eg, facilitator or administrator). At the completion of the survey, participants received a US $25 gift card for their participation.

### Measures

The 10-item System Usability Scale (SUS) is a tool for assessing the usability of a product (eg, websites, cell phones, and apps). The 10 items are scored on a 5-point Likert scale (range 0 *strongly disagree* to 4 *strongly agree*). A total usability score, representing a composite measure of usability is created by reversing the score of even-numbered items, summing the items, and multiplying by 2.5 to convert the original total scores of 0-40 to a 0-100 scale [28]. A score of 70 is considered average, 80 good, and 90 or above excellent usability [29,30]. The SUS is a simple and efficient tool for assessing the usability and user-friendliness of a technological platform with demonstrated reliability across multiple studies (α=.91) and strong evidence of a single factor structure [29]. Our administration of SUS reflected the revised language and overall rating of the user-friendliness introduced in Bangor and colleagues [30]. Survey responses were collected in REDCap [31,32].

Following completion of the SUS, participants were prompted to respond to open-ended questions to elicit further information on their opinions of the *ez*Dashboard. Questions included the frequency of use and the helpfulness of the *ez*Dashboard and items that may be missing or could be changed in future iterations. Facilitators were asked specifically how access to this real time information helps them work with parents using the *ez*Parent program and administrators were asked how the information helps them with overall management of the program. Finally, participants were asked to self-report their race or ethnicity, age, and gender.

### Analysis Plan

The composite SUS score was calculated by reverse scoring even-numbered items so that all items were scored in the same direction. Composite scores were then calculated by summing the item responses and multiplying them by 2.5 so that they fell on a scale of 0-100. Summary statistics were calculated for the composite scores and frequencies are reported for the overall rating of user-friendliness.

For analysis of the open-ended questions, 2 authors organized the responses by group (eg, administrators and facilitators) and conducted a thematic analysis of all responses based on the steps outlined by Braun and Clarke [33]. First, we reviewed all the responses and generated initial themes and categories. These categories were reviewed by the 2 authors and confirmed by a third author. Finally, we categorized the themes in order to provide a description and examples in this report. We quantified the comments in each category in order to provide a frequency related to participants’ ideas, suggestions, and ideas provided related to the usability of the *ez*Dashboard.

## Results

### Participants

A majority of participants (N=15) were women (n=12, 80%), White (n=11, 73%), with a mean age of 40.9 years (SD 13.9; range 20-68; Table 1). Participants were *ez*Parent facilitators (n=10) and administrators (n=4); 1 participant did not report their role with *ez*Parent. Participants (n=9) who were actively using the *ez*Dashboard at the time of the survey reported weekly use (n=3, 33%), once every few weeks (n=1, 11%), monthly (n=1, 11%), and less than monthly (n=4, 44%).

**Table 1:** Demographic characteristics of participants (N=15) enrolled in the *ez*Dashboard usability study.

Demographic characteristic		Participants, n (%)
**Gender**		
	Women	12 (80)
	Men	2 (13)
	Missing	1 (7)
**Race**		
	Black or African American	3 (20)
	More than 1 race^a^	1 (7)
	White	11 (73)
**Ethnicity**		
	Chose not to answer or missing	2 (13)
	Hispanic	0 (0)
	Not Hispanic	13 (87)

^a^In total, 1 participant reported their race as Black and other.

### About SUS

Scoring of the SUS indicated high usability of the *ez*Dashboard with a total mean score of 83.5 (range 47.5-100; SD 16.3; median 90, IQR 72.5-97.5). Overall, most participants (n=10, 67%) rated the overall user-friendliness of the *ez*Dashboard as *excellent* (n=9, 60%), or *best imaginable* (n=1, 7%; Table 2). On average new users (n=6) ranked the *ez*Dashboard higher (mean 90.8, SD 8.01) than existing users (n=9; mean 78.6, SD 18.84); however, this difference was not statistically significant (*P*=.16) and may be the result of high variability associated with small samples.

**Table 2:** Overall rating.

Overall user-friendliness rating	Participants, n (%)
Best imaginable	1 (7)
Excellent	9 (60)
Good	3 (20)
OK	2 (13)
Poor	0 (0)
Awful	0 (0)
Worst imaginable	0 (0)

### Open-Ended Survey Responses

#### Usefulness of the ezDashboard

Participants (N=15) reported that the dashboard allowed them to keep track of parent progress and identify potential problems that may arise with parent completion of *ez*Parent modules and serves as a helpful cue for discussion with parents. Specifically, 53% (8/15) of participants, both current users and participants who had not yet interacted with the *ez*Dashboard, identified that the *ez*Dashboard is or would be useful for monitoring parent progress and identifying trends in parent participation. As 1 facilitator reported, “I typically check it right before the calls to see the participant's last login and how much of the program they have completed.” Another reported, “This makes it much easier to track program adherence and help support parents who need a little extra help in completing the program.” Finally, a participant wrote, “Overall trends of participation are helpful to learn if any systemic barriers to participation need to be addressed.” In addition to monitoring progress, participants (n=5, 33%) identified the *ez*Dashboard as useful in reviewing topics for discussion and communicating with parents during the hybrid session. For example, 1 participant wrote “I would use the dashboard to...Refer back to specific content from modules,” and another “use it to inform the next group meeting, for example, if I noticed that everyone thought a previous module was difficult, I would give more time to that discussion.”

*ez*Parent administrators (n=4) identified that real-time data for *ez*Parent use helps overall management of program uptake and promotes parent motivation and accountability for module completion. For example, 1 program provides incentives for module completion and uses the *ez*Dashboard for tracking program use for the provision of these parent incentives. Administrators also provided that this information “would also be helpful for facilitators in learning when/how/if they need to modify their approach” when facilitating hybrid *ez*Parent and allows them to respond to questions from parents more promptly.

#### Suggested ezDashboard Changes

Participants (N=15) were asked if there was any information missing from the *ez*Dashboard (14 responded). Overall, participants suggested potential data points in the *ez*Dashboard to allow a more in-depth assessment of parent program use. Participants (n=4, 29%) identified that the ability to track partial progress through the modules would be useful, with suggestions of “it would be nice to see the # of logins for a week” and “it would be nice to differentiate if modules were completed or started, such as a sliding scale of how far through the module the parent has gotten so far.” In total, 2 (14%) participants suggested tracking the total time spent in each module as a method to understand meaningful engagement with the program and whether “a parent may be working a little too quickly through the program.” The administrative participants (n=4) believed “exportable, customizable reporting” would be useful for overall program management. In addition, more control in terms of modifying *ez*Dashboard information was identified, including amending parent information (eg, name and phone number) and the ability to group parents by cohort for tracking. In total, 38% (6/14) of the participants indicated that they would change nothing to make the *ez*Dashboard easier to use.

Beyond program metrics, participants identified changes that would integrate the hybrid delivery methods into the *ez*Dashboard. For example, including attendance records for hybrid meetings and integrating a method to directly communicate and contact parents (eg, texting) with parents in the dashboard so they could “nudge parent to modules.” Another suggestion included integrating the videoconference system into the dashboard so all program activities could occur in 1 place.

## Discussion

### Principal Findings: ezDashboard Usability

The purpose of this usability study was to identify the overall performance of the *ez*Parent administrative dashboard and understand users’ perceptions of the ease of navigation and usefulness of the *ez*Dashboard in implementing hybrid delivery of the *ez*Parent program. Further, we were interested in collecting users’ suggestions for changes or additions to the *ez*Dashboard to improve the overall user experience for real-world use.

Overall, ratings on the SUS indicate good usability (mean 83.5, SD 16.3; median 90, IQR 72.5-97.5). According to Bangor and colleagues [29], a mean score of 83.5 is in the fourth quartile of scores, rated as acceptable, and falls between the good to excellent range using an adjective rating scale. In addition, overall ratings of the user-friendliness of the *ez*Dashboard among all participants were positive (*ok, good, excellent,* and *best imaginable*). These initial ratings are promising, show an acceptable level of usability, and the written feedback provides us with concrete methods for improving the *ez*Dashboard.

This study’s participants reported variable use of the *ez*Dashboard (eg, ranging from weekly to less than monthly). The differences in use may be a function of the individual’s role using *ez*Parent (eg, administrators may only need to use the dashboard monthly for overall program management while a coach conducting weekly calls would use it weekly). We do, however, need to consider the variability of use as a potential function of the overall usefulness of the information provided in the *ez*Dashboard. Therefore, our next steps will be to provide clear instructions and descriptions of *ez*Dashboard use as well as integrate suggested changes. Changes to the *ez*Dashboard to provide desired information of parent use may increase regular use and uptake.

Harrington and colleagues [10] highlight the importance of including program usage metrics in dashboards to allow interventionists to make data-driven decisions, identify areas for program improvement, and support the ability to provide personalized support to program users. Further, real-time data can support tailored approaches to increase program uptake. Overall, our participants reported that they were able to use the *ez*Dashboard information to take an individualized approach to the hybrid delivery of the *ez*Parent program, and administrators used the *ez*Dashboard data to provide oversight and incentives to parent participants. In addition, there were several excellent suggestions for *ez*Dashboard improvements. The suggested changes varied by participant role with *ez*Parent.

Facilitator suggestions were primarily fine-tuning the data presented in the *ez*Dashboard to provide more nuanced use metrics beyond module completion. Since most digital analytics provide summary statistics, the ability to gather use metrics at the individual level provides important information for the facilitation of program engagement [14]. The data points suggested were partial module completion, identifying the actual location within the module of last use, and average time spent on the page. While the *ez*Dashboard currently provides time stamps for date and time, the modules were completed and allows for a rough estimate of the speed at which a parent is moving through the program; more specific analytics for time in the program could provide more meaningful estimates for engagement. In our previous work, we found that on average parents spent 37.2 (SD 22.2) minutes per module with a range from 26.4 (module 5) to 47.9 (module 2) minutes [21]. Variations across modules occurred because of variations in pages per module; however, there was a significant decrease in minutes/module over time (eg, participants spent less time on later modules) [21]. This information is important for facilitators to monitor parent engagement and to encourage active involvement in the modules, particularly in later modules when data support a decrease in overall time of engagement.

The administrative participants identified other types of metrics and functions that would be helpful for program management (eg, the ability to download individual and cohort reports of usage metrics to allow for more streamlined monitoring and administrative access to modify participant demographics). The goal of the *ez*Dashboard is to present individual use data and adaptations to include monitoring cohorts, which could increase administrative efficiencies. A benefit of web-based interventions is the reach and accessibility of programs and the integration of administrator dashboards for monitoring and management and has the potential to increase program uptake and overall efficiencies in program delivery. Future evaluation of the *ez*Dashboard will focus on the effects of *ez*Dashboard use on implementation factors related to organizational and individual program uptake and delivery.

### Limitations

Although deemed sufficient for usability testing to acquire feedback on user experience, our sample size was small. This study was an initial evaluation of the *ez*Dashboard and provides valuable information for modifications. Our next steps are to integrate these findings into the *ez*Dashboard to evaluate overall use of *ez*Parent and dashboard use in a pragmatic trial with a larger sample of facilitators and administrators.

Additional limitations of this study include the usability testing being done virtually and at only 1 timepoint, which precluded our ability to examine participants’ actions in real time while using the *ez*Dashboard in a pragmatic ongoing setting. Users’ feedback may change after continued use of the *ez*Dashboard. The administrative dashboard was only tested for 1 specific intervention, *ez*Parent; however, we believe these results could be applicable and inform the development of dashboards for different types of web-based programs.

### Conclusions

The *ez*Dashboard was initially developed to provide individual parent usage data to facilitators in real time to monitor parent progress in the program and support parent program uptake. The results of usability testing indicate that the *ez*Dashboard is easy to use and provides functional information to facilitators in delivering the *ez*Parent intervention. Providing resources for facilitators and administrators to aid in facilitation of the hybrid intervention may lead to improved parent uptake and outcomes [34]. Qualitative results indicate that integrating suggested features into the *ez*Dashboard may help provide a smoother experience for facilitators, administrators, and ultimately the parents using the program. Administrative dashboards that provide real-time program usage data require an investment in the upfront cost of program development. The user facing program, in this case the *ez*Parent, must be built to collect the user data that are to be displayed in the dashboard. For those considering integrating a dashboard for a web-based intervention, we suggest early planning during the initial development.

## Abbreviations

CPP: Chicago Parent Program
PT: parent training
RCT: randomized controlled trial
REDCap: Research Electronic Data Capture
SUS: System Usability Scale

## Data Availability

The data sets generated and analyzed during this study are available from the corresponding author upon reasonable request. Generative artificial intelligence was not used in any portion of this paper’s writing.
